# Steatotic liver disease subtypes and risk of hospitalization for sepsis: a nationwide cohort study

**DOI:** 10.1080/07853890.2026.2704290

**Published:** 2026-07-25

**Authors:** Hye Seong, Se Yun Kim, Eun Hwa Lee, Dayeong Kim, Kyung Do Han, Sang Hoon Han

**Affiliations:** ^a^ Division of Infectious Diseases, Department of Internal Medicine, Yonsei University College of Medicine, Seoul, Republic of Korea; ^b^ Department of Digital Health, SAIHST, Sungkyunkwan University, Seoul, Republic of Korea; ^c^ Department of Statistics and Actuarial Science, Soongsil University, Seoul, Republic of Korea; ^d^ Institute for Innovation in Digital Healthcare, Yonsei University College of Medicine, Seoul, Republic of Korea

**Keywords:** Steatotic liver disease, metabolic dysfunction-associated steatotic liver disease, metabolic dysfunction and alcohol-related liver disease, alcohol-related liver disease, sepsis, nationwide cohort

## Abstract

**Background:**

The recent reclassification of steatotic liver disease (SLD) into metabolic dysfunction-associated SLD (MASLD), MASLD with increased alcohol intake (MetALD), and alcohol-related liver disease (ALD) provides a clinically relevant framework for distinguishing heterogeneous etiologic subtypes. However, whether these newly defined SLD subtypes differ in their susceptibility to sepsis remains unclear. We assessed the incidence and risk of hospitalization for sepsis across the SLD spectrum in a nationwide cohort.

**Methods:**

We conducted a retrospective cohort study using the Korean National Health Insurance Service database, including 4,389,734 adults who underwent health screening in 2012. SLD was defined as a fatty liver index (FLI) ≥30 and subtyped as MASLD, MetALD, or ALD based on cardiometabolic risk profiles and alcohol intake; FLI <30 defined the non-SLD reference group. Sepsis was identified using hospitalization claims and ICD-10 codes from 2013 to 2022. Adjusted hazard ratios (aHRs) were estimated using multivariable Cox proportional hazards models, with subgroup and sensitivity analyses.

**Results:**

The cohort comprised MASLD (*n* = 1,332,944; 30.4%), MetALD (*n* = 164,387; 3.7%), and ALD (*n* = 79,445; 1.8%). Sepsis incidence per 1,000 person-years was 3.11, 2.25, and 4.49, respectively, compared with 2.20 in the non-SLD group. After adjusting for established sepsis risk factors, all subtypes remained associated with higher risk, greatest in ALD (aHR 1.53), followed by MetALD (1.11) and MASLD (1.07) (all *p* < 0.001). MetALD showed lower crude incidence than MASLD but a higher adjusted risk in multivariable models. In sensitivity analyses restricted to individuals without comorbidities, associations persisted: ALD (aHR 1.60), MetALD (1.16), and MASLD (1.09) (all *p* < 0.001).

**Conclusions:**

All SLD subtypes were associated with increased but distinct risks of hospitalization for sepsis, with ALD conferring the highest risk. These findings support subtype-specific risk stratification and may inform infection-prevention strategies and clinical surveillance in individuals with SLD.

## Introduction

Sepsis represents a major clinical challenge in chronic liver disease, where complex liver–immune interactions contribute to excess morbidity and mortality despite advances in critical care [1,2]. Among chronic liver diseases, cirrhosis confers the highest risk of infection and sepsis-related mortality, followed by severe alcoholic hepatitis, while viral hepatitis and autoimmune liver diseases typically confer a lower risk unless they have progressed to cirrhosis [3]. Building on this spectrum of risk, recent evidence, predominantly from studies on non-alcoholic fatty liver disease (NAFLD), suggests that patients with fatty liver disease may also be susceptible to sepsis and infection-related adverse outcomes [1,4,5].

In 2023, an international consensus panel redefined NAFLD by introducing the concept of steatotic liver disease (SLD) as an overarching umbrella category. This new framework addresses several limitations of the previous nomenclature – including its reliance on exclusion-based diagnostic criteria, insufficient recognition of metabolic dysfunction, and concerns regarding stigma – while providing a unified conceptual structure that encompasses the diverse etiologies of SLD [6]. The revised SLD classification delineates a spectrum of distinct subtypes – metabolic dysfunction-associated SLD (MASLD), metabolic dysfunction and alcohol-related liver disease (MetALD), defined as MASLD with increased alcohol intake, alcohol-related liver disease (ALD), cryptogenic SLD, and SLD of specific etiologies – thereby highlighting the overlapping but etiologically distinct pathways contributing to steatotic liver disease [6,7].

SLD is characterized by the predominant influence of metabolic dysfunction, alcohol consumption, or both. Its three major etiologic categories – MASLD, MetALD, and ALD – share progressive hepatocellular injury and chronic inflammation, but arise from partly distinct metabolic, alcohol-related, and immune-mediated mechanisms. These pathophysiological processes may collectively increase vulnerability to severe infection and sepsis [1,2]. Given that sepsis remains a life-threatening condition with limited targeted therapeutic options beyond early recognition, source control, antimicrobial treatment, and supportive care [8,9], accurately identifying which SLD subtypes confer the greatest susceptibility to severe infection is clinically important. Such insights may enhance risk stratification, support preventive strategies, and promote early recognition of infection-related deterioration.

Although previous studies have separately investigated the associations of NAFLD, metabolic syndrome, and alcohol-related liver injury with sepsis [2,4, 10–12], the comparative risk of sepsis across the newly defined SLD subtypes – particularly MASLD and MetALD – remains poorly understood. To date, large-scale population-based evidence comparing sepsis risk across these reclassified SLD categories is lacking.

In this nationwide, long-term cohort study, we aimed to quantify the incidence and risk of hospitalization for sepsis across MASLD, MetALD, and ALD subtypes while accounting for demographic, lifestyle, and clinical confounders. By comparing sepsis risk across etiologically distinct SLD subtypes, this study may help inform subtype-specific risk assessment and infection-prevention strategies in clinical practice.

## Materials and methods

### Study design and data source

This retrospective longitudinal cohort study used data from the Korean National Health Insurance Service (NHIS), which provides compulsory, nationwide health coverage for the entire South Korean population. All individuals aged ≥20 years are eligible for and routinely invited to undergo the biennial National Health Screening Program (NHSP), which collects standardized clinical, lifestyle, and anthropometric data (Table S1) [13]. The NHSP data are systematically linked to the NHIS administrative claims database to form the NHIS–National Health Screening Cohort (NHIS–HEALS), enabling longitudinal assessment of healthcare utilization and clinical outcomes, including outpatient visits and hospital admissions.

This study was designed to estimate the association between SLD subtypes and subsequent hospitalization for sepsis using routinely collected nationwide health screening and claims data. The operational definitions and classification criteria were selected to align with recent SLD nomenclature while remaining applicable to population-based health screening data.

This study was approved by the Institutional Review Board of Gangnam Severance Hospital, Yonsei University College of Medicine (IRB No. 3-2023-0267), with a waiver of informed consent. After approval by the Healthcare Big Data Open System (study ID: REQ2025040336), the Korean NHIS data were provided in a fully anonymized form. The study was conducted in accordance with the principles of the Declaration of Helsinki.

### Population-based cohort selection

Using an age- and sex-stratified random sampling approach, we selected 40% of individuals from the 2012 NHIS-HEALS cohort, resulting in an initial cohort of 4,910,068 individuals drawn from the 12,275,000 adults aged ≥20 years who participated in the 2012 NHSP. We then excluded individuals with a history of malignant neoplasm of the liver and intrahepatic bile ducts, defined by International Classification of Diseases, 10th Revision (ICD-10) code C22 or the Korean rare intractable disease registration code V193, a government-issued identifier for patients with cancer receiving special insurance benefits. Individuals with a history of liver transplantation (ICD-10: Z94.4) and those with other chronic liver diseases, including viral hepatitis, autoimmune hepatitis, drug-induced liver disease, and hemochromatosis, were also excluded, with corresponding ICD-10 codes provided in Table S2.

**Figure 1.**
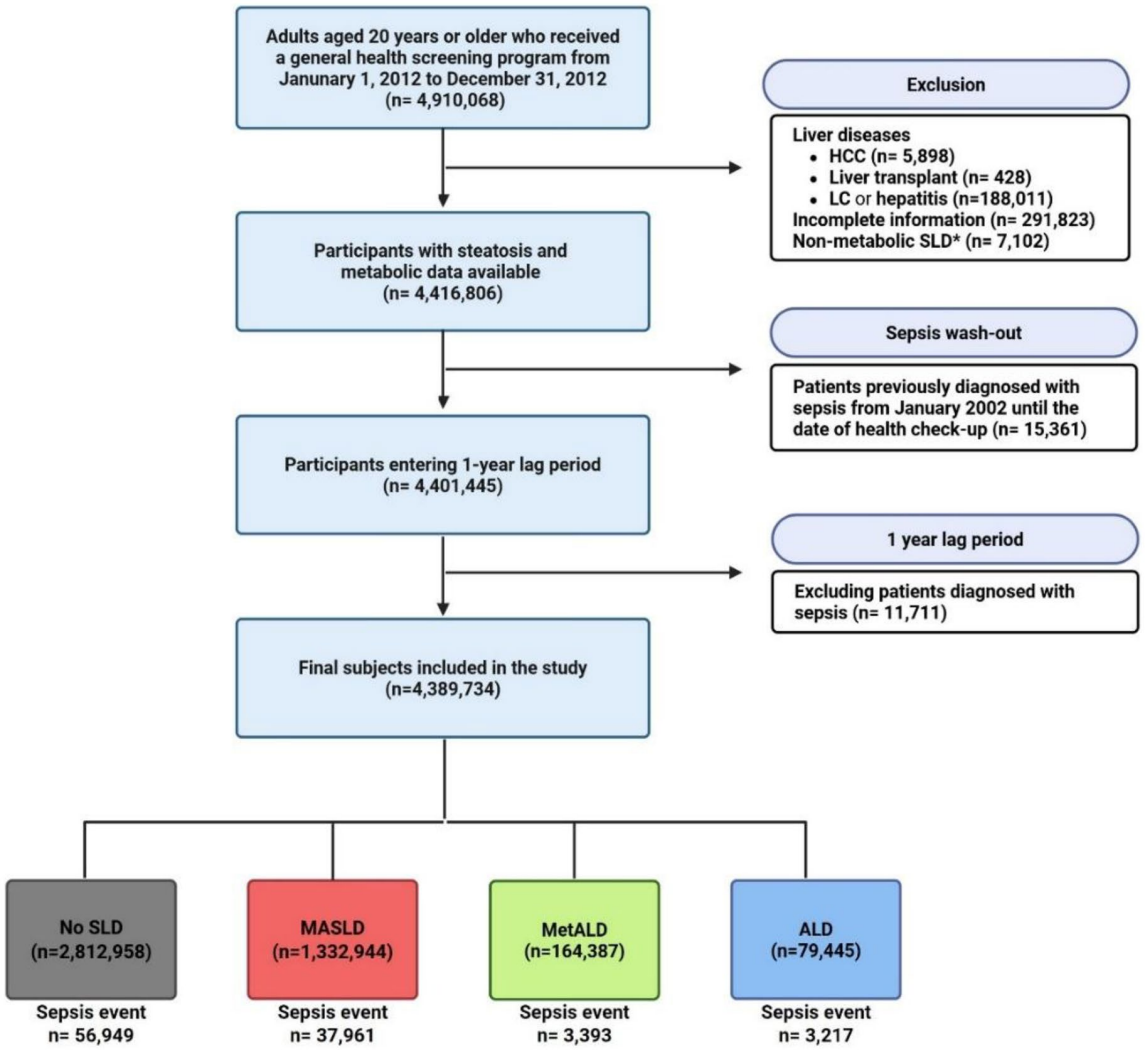
Flowchart of the study population selection. Selection of the study population, including classification into SLD subtypes or Non-SLD, after excluding individuals with liver diseases, incomplete baseline data, non-metabolic SLD, or a prior history of sepsis. The final cohort was followed until sepsis occurrence, death, or December 31, 2022. *Non-metabolic SLD, also referred to as cryptogenic SLD, was defined as a fatty liver index ≥30 in the absence of any cardiometabolic risk factors, based on MASLD adult diagnostic criteria of the Asia–Pacific region. Abbreviations: ALD: alcohol-related liver disease; HCC: hepatocellular carcinoma; LC: liver cirrhosis; MASLD: metabolic dysfunction-associated steatotic liver disease; MetALD: metabolic dysfunction and alcohol-related liver disease; SLD: steatotic liver disease.

Individuals with missing baseline data or cryptogenic SLD – defined as FLI ≥30 in the absence of cardiometabolic risk factors (CMRFs) and without any other identifiable liver disease – were excluded [6,14]. Additionally, individuals with a diagnosis of sepsis during the one-year washout period before health screening were excluded. To minimize reverse causation, we further excluded individuals who developed sepsis during the one-year lag period after the health screening date, resulting in a final analytic cohort of 4,389,734 individuals (Figure 1).

### Classification of SLD subtypes

We calculated the FLI using laboratory and anthropometric measurements obtained from the NHSP and operationally defined SLD as an FLI ≥30. This threshold was selected based on validation data from asymptomatic Korean adults, in whom an FLI cutoff of 29 was optimal for identifying ultrasound-defined NAFLD [15]. SLD subtypes were classified using an operational definition based on the 2023 international consensus statement [6], incorporating FLI, alcohol intake, and CMRFs.

Individuals were categorized as follows: (1) MASLD, defined as FLI ≥30 in the presence of at least one CMRF and mean alcohol consumption <30 g/day for men or <20 g/day for women; (2) MetALD, defined as meeting MASLD criteria with mean alcohol consumption of 30–60 g/day for men or 20–50 g/day for women; and (3) ALD, defined as meeting MASLD criteria with either mean alcohol consumption ≥60 g/day for men or ≥50 g/day for women, or the presence of diagnostic codes for ALD or alcohol abuse/misuse, as detailed in Table S2. Individuals with FLI <30 were classified as the non-SLD group and served as the reference category [6,7].

The CMRFs were defined using Asian-specific criteria for body mass index (≥23 kg/m^2^) and waist circumference (>90 cm for men and >85 cm for women) [16]. Two-hour post-load glucose and HbA1c levels were not assessed in the NHSP and were therefore not included in the operational criteria. All other components were defined according to the consensus statement [6].

### Primary clinical outcome

The primary outcome was incident sepsis, defined as the first hospitalization with explicit ICD-10 diagnostic codes for sepsis-related conditions, including septic shock (R57.2) and systemic inflammatory response syndrome of infectious origin, without or with organ failure (R65.0 and R65.1) (Table S3) [17]. Although claims-based ICD-10 codes were used, the outcome definition was restricted to hospitalized cases to capture clinically severe events. To minimize misclassification, cases recorded only in outpatient settings, as well as neonatal sepsis (P36) and puerperal sepsis (O85), were excluded.

Follow-up commenced one year after the health screening date and continued until the first occurrence of sepsis, death, or December 31, 2022, whichever came first. The one-year lag period was applied to reduce the likelihood that undiagnosed or pre-existing severe illness influenced both baseline SLD classification and subsequent sepsis occurrence. The mean follow-up duration was approximately nine years (Figure 1).

### Clinical variables and risk factors

Covariates were obtained from health screening measurements and claims data recorded at baseline in the NHIS–HEALS database. Low-income status was defined as being a recipient of the national Medical Aid program, which provides government-funded healthcare coverage for low-income households, or as belonging to the lowest quartile of household income according to the 2010 Korean National Housing Census.

Body mass index (BMI) was classified into five categories according to Asia-Pacific and Korean guidelines: <18.5 kg/m^2^, 18.5–22.9 kg/m^2^, 23.0–24.9 kg/m^2^, 25.0–29.9 kg/m^2^, and ≥30.0 kg/m^2^ (Table S4) [16]. Laboratory parameters included fasting glucose, total cholesterol, high-density lipoprotein cholesterol, low-density lipoprotein cholesterol, triglycerides, estimated glomerular filtration rate, aspartate aminotransferase, alanine aminotransferase, and gamma-glutamyl transferase. All blood samples were obtained after an overnight fast of at least 12 h.

Lifestyle factors included smoking status, alcohol intake, and physical activity. Smoking status was categorized as never, former, or current smoker. Average alcohol consumption was assessed using a standardized health screening questionnaire, in which individuals reported their usual daily intake as the number of drinks consumed. Alcohol intake was standardized by converting one standard drink to 10 g of absolute alcohol, in accordance with World Health Organization guidelines [18]. Individuals were categorized as nondrinkers (0 g/day), low drinkers (<30 g/day for men and <20 g/day for women), moderate drinkers (30–60 g/day for men and 20–50 g/day for women), and heavy drinkers (≥60 g/day for men and ≥50 g/day for women), according to international criteria [7,18] (Table S4). Regular physical activity was defined as moderate-intensity exercise on at least five days per week or vigorous-intensity exercise on at least three days per week, based on standardized self-reported questionnaires [19,20].

Comorbidities were defined using ICD-10 diagnostic codes combined with relevant clinical criteria and prescription data. Type 2 diabetes mellitus was defined as codes E11–E14 with antidiabetic medication use or fasting glucose ≥126 mg/dL; hypertension as codes I10–I13 or I15 with antihypertensive medication use, systolic blood pressure ≥140 mmHg, or diastolic blood pressure ≥90 mmHg; dyslipidemia as code E78 with lipid-lowering medication use or total cholesterol ≥240 mg/dL; and chronic kidney disease as estimated glomerular filtration rate <60 mL/min/1.73 m^2^ or a history of end-stage renal disease identified by rare intractable disease registration codes in the Korean NHIS, including V001 for hemodialysis, V003 for peritoneal dialysis, and V005 for post-kidney transplantation care involving immunosuppressive therapy (Table S4).

The Charlson Comorbidity Index (CCI) was calculated from 17 ICD-10-defined conditions using the Quan et al. algorithm [21], with weights assigned according to 1-year mortality risk and age excluded; scores were categorized as 0, 1, or ≥2 (Table S5). To adjust for established risk factors for sepsis, analyses also accounted for immunocompromised status, including solid organ transplantation other than liver transplantation, hematologic malignancy, solid cancer, HIV infection, or hyposplenism, as well as chronic kidney, heart, and lung diseases (Table S6).

### Statistical analysis

Baseline characteristics were summarized as means with standard deviations for continuous variables and frequencies with percentages for categorical variables. Because of the very large sample size, between-group differences were expected to be statistically significant even for small absolute differences; therefore, clinical relevance was considered when interpreting baseline characteristics. Differences among SLD subtypes were compared using one-way analysis of variance for continuous variables and the χ^2^ test for categorical variables.

Sepsis incidence rates were calculated per 1,000 person-years. Cox proportional hazards regression was used to estimate hazard ratios (HRs) and 95% confidence intervals (CIs) for the association between SLD subtype and incident hospitalization for sepsis, using the non-SLD group as the reference category.

Regression models were constructed sequentially. Model 1 was unadjusted. Model 2 was adjusted for age and sex. Model 3 was adjusted for age, sex, income, smoking status, regular physical activity, and CCI score. Model 4 was adjusted for age, sex, income, smoking status, regular physical activity, type 2 diabetes mellitus, hypertension, dyslipidemia, chronic kidney disease, chronic heart disease, chronic lung disease, and immunocompromised status. The proportional hazards assumption was verified using Schoenfeld residuals, and no significant violations were observed.

Cumulative incidence of sepsis was estimated for each SLD subtype using the Kaplan–Meier method, with differences across groups assessed using the log-rank test. Time-to-event was defined as the interval from the start of follow-up, one year after the baseline health screening date, to the first occurrence of sepsis requiring hospitalization, with censoring at death or the end of the study period, whichever occurred first.

In addition to the main multivariable models, we performed six sensitivity analyses. First, to assess the robustness of the findings to the selected FLI threshold, we repeated the primary Cox proportional hazards analyses by replacing the FLI ≥30 threshold in the primary SLD classification algorithm with FLI ≥60; all other subtype criteria and covariate adjustment models were unchanged. Second, to evaluate the potential influence of alcohol-related risk among individuals classified as non-SLD, we repeated the analyses after excluding individuals with FLI <30 who met the heavy alcohol intake criterion or had ICD-10 codes for ALD or alcohol abuse/misuse. Third, to evaluate the influence of including systemic inflammatory response syndrome without organ failure in the primary outcome, we repeated the analyses after removing ICD-10 code R65.0 from the outcome code set. Fourth, to account for the competing risk of death, we performed Fine–Gray subdistribution hazards regression, treating death before hospitalization for sepsis as a competing event. This analysis was conducted using the EVENTCODE option in PROC PHREG in SAS, and the results are reported as subdistribution hazard ratios (sHRs) with 95% confidence intervals. Fifth, to evaluate whether the findings were sensitive to the length of follow-up in the context of potential changes in SLD status over time, we repeated the primary analyses while restricting follow-up to the first 3 and 5 years after follow-up initiation. Finally, we restricted the analysis to individuals with a CCI score of 0 to assess whether the observed associations persisted among individuals without baseline comorbidity burden. All analyses were performed using SAS 9.4 (SAS Institute, Cary, NC, USA), with two-sided *p* < 0.05 considered statistically significant.

## Results

### Clinical characteristics and risk profiles

Among the study population, 2,812,958 individuals (64.1%) had no evidence of SLD, while 1,332,944 (30.4%) met criteria for MASLD, 164,387 (3.7%) for MetALD, and 79,445 (1.8%) for ALD. The mean age of the cohort was 48.1 years, and 53.9% were male. Individuals in the MASLD (50.1 years) and ALD (50.0 years) groups were older than the cohort average, whereas those in the MetALD (46.1 years) and non-SLD (47.2 years) groups were younger. The proportion of individuals aged 40–64 years was lowest in the non-SLD group (57.2%) and higher in the MASLD (62.1%), MetALD (65.0%), and ALD (69.0%) groups. The non-SLD group was the only group in which females outnumbered males, with women accounting for 58.5% of the group. In contrast, men accounted for the majority of each SLD subtype, comprising 73.0% of the MASLD group, 94.4% of the MetALD group, and 91.2% of the ALD group (Table 1).

**Table 1.** Baseline characteristics of the study population according to steatotic liver disease subtype.

Characteristics	Total individuals (*n* = 4,389,734)	Groups				*P*-value
		Non-SLD (*n* = 2,812,958)	MASLD (*n* = 1,332,944)	MetALD (*n* = 164,387)	ALD (*n* = 79,445)	
Age, years	48.11 ± 13.79	47.23 ± 14.16	50.08 ± 13.11	46.13 ± 11.37	50.01 ± 12.03	<0.001
Age groups, years						<0.001
< 40	1,222,636 (27.85)	853,068 (30.33)	306,768 (23.01)	47,434 (28.86)	15,366 (19.34)	
40–64	2,599,004 (59.21)	1,609,813 (57.23)	827,483 (62.08)	106,915 (65.04)	54,793 (68.97)	
≥ 65	568,094 (12.94)	350,077 (12.45)	198,693 (14.91)	10,038 (6.11)	9,286 (11.69)	
Sex, male	2,367,364 (53.93)	1,166,466 (41.47)	973,326 (73.02)	155,139 (94.37)	72,433 (91.17)	<0.001
BMI, kg/m^2^	23.76 ± 3.28	22.24 ± 2.41	26.54 ± 2.86	25.97 ± 2.88	25.96 ± 3.06	<0.001
BMI groups, kg/m^2^						<0.001
<18.5	160,860 (3.66)	159,301 (5.66)	805 (0.06)	393 (0.24)	361 (0.45)	
18.5–22.9	1,700,809 (38.75)	1,573,489 (55.94)	96,711 (7.26)	19,586 (11.91)	11,023 (13.88)	
23.0–24.9	1,072,656 (24.44)	713,883 (25.38)	297,861 (22.35)	41,957 (25.52)	18,955 (23.86)	
25.0–29.9	1,283,198 (29.23)	362,703 (12.89)	790,222 (59.28)	88,510 (53.84)	41,763 (52.57)	
≥30	172,211 (3.92)	3,582 (0.13)	147,345 (11.05)	13,941 (8.48)	7,343 (9.24)	
Waist circumference, cm	80.23 ± 9.28	75.67 ± 7.08	88.39 ± 6.82	88.04 ± 6.97	88.45 ± 7.35	<0.001
Smoking						<0.001
Never	2,625,308 (59.81)	1,961,001 (69.71)	617,444 (46.32)	28,029 (17.05)	18,834 (23.71)	
Former	682,266 (15.54)	331,713 (11.79)	284,453 (21.34)	44,754 (27.22)	21,346 (26.87)	
Current	1,082,160 (24.65)	520,244 (18.49)	431,047 (32.34)	91,604 (55.72)	39,265 (49.42)	
Alcohol drinking^a^						<0.001
None	2,228,238 (50.76)	1,614,708 (57.40)	602,707 (45.22)	0 (0)	10,823 (13.62)	
Low	1,812,727 (41.29)	1,063,517 (37.81)	730,237 (54.78)	0 (0)	18,973 (23.88)	
Moderate	286,837 (6.53)	114,935 (4.09)	0 (0)	164,387 (100)	7,515 (9.46)	
Heavy	61,932 (1.41)	19,798 (0.70)	0 (0)	0 (0)	42,134 (53.04)	
Regular exercise^b^	839,085 (19.11)	540,271 (19.21)	249,601 (18.73)	32,546 (19.8)	16,667 (20.98)	<0.001
Income, lowest quartile	816,036 (18.59)	558,185 (19.84)	221,833 (16.64)	21,786 (13.25)	14,232 (17.91)	<0.001
Co-morbidities						
T2DM	426,028 (9.71)	173,118 (6.15)	211,533 (15.87)	22,862 (13.91)	18,515 (23.31)	<0.001
Hypertension	1,185,240 (27.00)	547,635 (19.47)	534,749 (40.12)	63,591 (38.68)	39,265 (49.42)	<0.001
SBP, mmHg	121.94 ± 14.79	118.82 ± 14.21	127.27 ± 14.14	128.81 ± 14.05	129.09 ± 14.39	<0.001
DBP, mmHg	76.08 ± 9.98	74.03 ± 9.50	79.49 ± 9.70	81.29 ± 9.94	80.95 ± 9.93	<0.001
Dyslipidemia	881,977 (20.09)	413,656 (14.71)	402,197 (30.17)	39,293 (23.90)	26,831 (33.77)	<0.001
Chronic kidney disease	163,114 (3.72)	90,431 (3.21)	66,494 (4.99)	3,367 (2.05)	2,822 (3.55)	<0.001
Chronic heart disease	305,259 (6.95)	160,114 (5.69)	126,290 (9.47)	9,569 (5.82)	9,286 (11.69)	<0.001
Chronic lung disease	497,415 (11.33)	313,089 (11.13)	160,872 (12.07)	13,795 (8.39)	9,659 (12.16)	<0.001
At least one CMRF	3,575,199 (81.44)	1,998,423 (71.04)	1,332,944 (100)	164,387 (100)	79,445 (100)	<0.001
Immunocompromised state	111,100 (2.53)	74,408 (2.65)	32,523 (2.44)	1,915 (1.16)	2,254 (2.84)	<0.001
Transplant status^c^	1,332 (0.03)	915 (0.03)	404 (0.03)	7 (0)	6 (0.01)	<0.001
Hematologic malignancy	2,489 (0.06)	1,524 (0.05)	906 (0.07)	35 (0.02)	24 (0.03)	<0.001
Solid cancer	107,290 (2.44)	72,020 (2.56)	31,188 (2.34)	1,860 (1.13)	2,222 (2.8)	<0.001
Immunodeficiency, HIV	531 (0.01)	288 (0.01)	218 (0.02)	17 (0.01)	8 (0.01)	<0.001
CCI score						<0.001
0	2,194,543 (49.99)	1,460,886 (51.93)	620,443 (46.55)	88,505 (53.84)	24,709 (31.10)	
1	996,636 (22.70)	633,377 (22.52)	306,602 (23.00)	37,888 (23.05)	18,769 (23.63)	
≥ 2	1,198,555 (27.30)	718,695 (25.55)	405,899 (30.45)	37,994 (23.11)	35,967 (45.27)	
Laboratory test						
Glucose, mg/dL	97.66 ± 22.93	94.00 ± 18.38	103.76 ± 27.81	105.22 ± 28.37	109.47 ± 33.05	<0.001
Total C, mg/dL	194.97 ± 36.53	189.58 ± 34.53	204.98 ± 37.98	203.77 ± 37.13	199.40 ± 39.47	<0.001
HDL-C, mg/dL	55.55 ± 17.56	58.59 ± 17.9	49.56 ± 15.24	53.37 ± 17.04	53.01 ± 16.11	<0.001
LDL-C, mg/dL	114.11 ± 33.99	112.17 ± 31.75	118.87 ± 37	111.71 ± 38.17	107.85 ± 39.84	<0.001
AST, IU/L	23.24 (23.23–23.24)	21.37 (21.36–21.38)	26.45 (26.44–26.47)	29.19 (29.13–29.25)	31.78 (31.67–31.89)	<0.001
ALT, IU/L	20.73 (20.72–20.74)	17.07 (17.06–17.08)	29.02 (28.99–29.05)	30.61 (30.54–30.69)	31.81 (31.68–31.93)	<0.001
GGT, IU/L	25.77 (25.75–25.79)	18.65 (18.64–18.67)	42.18 (42.13–42.22)	70.27 (70.04–70.51)	77.10 (76.67–77.54)	<0.001
Triglyceride, mg/dL	109.6 (109.5–109.6)	85.3 (85.2–85.3)	169.9 (169.7–170.0)	181.6 (181.2–182.1)	179.0 (178.3–179.6)	<0.001
eGFR, mL/min/1.73m^2^	91.92 ± 35.57	93.10 ± 34.66	89.31 ± 37.30	92.73 ± 36.00	92.03 ± 34.44	<0.001
Sepsis	**101,520 (2.31)**	**56,949 (2.02)**	**37,961 (2.85)**	**3,393 (2.06)**	**3,217 (4.05)**	**<0.001**

All data are presented as mean ± standard deviation or geometric mean (95% CI) or number (percent). ^a^Alcohol consumption was classified according to the average daily drinking habit; (1) none, 0 g/day, (2) low, <30 g/day for men and <20 g/day for women, (3) moderate, 30–60 g/day for men and 20–50 g/day for women, and (4) heavy, ≥60 g/day for men and ≥50 g/day for women. ^b^Defined as moderate-intensity exercise for ≥ 5 days a week or vigorous-intensity exercise for ≥ 3 days in a week on last 7-day self-report questionnaire. ^c^Transplant status includes solid organ transplantation other than liver transplantation and hematopoietic stem cell transplantation.

Abbreviations: ALD: alcohol-related liver disease; ALT: alanine aminotransferase; AST: aspartate transaminase; BMI: body mass index; CCI: Charlson Comorbidity Index; CMRF: cardiometabolic risk factor; DBP: diastolic blood pressure; eGFR: estimated glomerular filtration rate; GGT: gamma-glutamyl transferase; HDL-C: high-density lipoprotein-cholesterol; HIV: human immunodeficiency virus; IU: international unit; LDL-C: low density lipoprotein-cholesterol; MASLD: metabolic dysfunction-associated steatotic liver disease; MetALD: metabolic dysfunction and alcohol-related liver disease; SBP: systolic blood pressure; SLD: steatotic liver disease; T2DM: type 2 diabetes mellitus; Total C: total cholesterol.

The MASLD group showed the highest mean BMI (26.5 kg/m^2^) and obesity prevalence, defined as BMI ≥25 kg/m^2^, at 70.3%, whereas the non-SLD group had a substantially lower obesity prevalence of 13.0%. Never-smoking was most common in the non-SLD group (69.7%), while current smoking was most frequent in the MetALD group (55.7%). As expected from the subtype definitions, no individuals in the MASLD group met criteria for moderate or heavy alcohol consumption, whereas all individuals in the MetALD group were classified as moderate drinkers. Only 4.8% of individuals in the non-SLD group reported moderate or greater alcohol consumption, whereas 53.0% of those in the ALD group met the criteria for heavy drinking, consistent with the alcohol-based classification of ALD. Among the 79,445 individuals classified as having ALD, 39,307 (49.48%) were identified based on heavy alcohol intake alone, 37,311 (46.96%) based on ICD-10 codes for ALD or alcohol abuse/misuse alone, and 2,827 (3.56%) based on both criteria. Overall, 42,134 individuals (53.04%) met the heavy alcohol intake criterion, and 40,138 (50.52%) had relevant ICD-10 codes, with some overlap between the two criteria.

### Sepsis incidence and risk across SLD subtypes

During the 9-year follow-up period, 101,520 incident sepsis cases were identified, corresponding to 2.31% of the total cohort. The proportion of individuals who experienced a first hospitalization for sepsis was highest in the ALD group (4.05%) and lowest in the non-SLD group (2.02%) (overall *p* < 0.001) (Table 1). The incidence rate of sepsis in the MetALD group was 2.25 per 1,000 person-years, which was comparable to that in the non-SLD group (2.20 per 1,000 person-years). Higher incidence rates were observed in the MASLD and ALD groups, at 3.11 and 4.49 per 1,000 person-years, respectively (Table 2).

**Table 2.** Sepsis incidence and risk estimates by SLD subtype, unadjusted and adjusted.

SLD subtypes	Sepsis^a^	Duration of follow-up (years)	IR^b^	HR (95% CI)			
				Unadjusted Model 1	Adjusted model 2^c^	Adjusted model 3^d^	Adjusted model 4^e^
Non-SLD	56,949	25,918,127	2.20	1 (Ref.)	1 (Ref.)	1 (Ref.)	1 (Ref.)
MASLD	37,961	12,203,059	3.11	1.420 (1.402–1.439)	1.196 (1.180–1.212)	1.134 (1.119–1.149)	1.071 (1.056–1.086)
MetALD	3,393	1,509,863	2.25	1.028 (0.993–1.064)	1.259 (1.216–1.305)	1.172 (1.131–1.214)	1.108 (1.069–1.148)
ALD	3,217	715,994	4.49	2.052 (1.981–2.127)	1.838 (1.773–1.905)	1.524 (1.470–1.580)	1.530 (1.475–1.587)

^a^Refers to the first episode of sepsis requiring hospitalization. ^b^Per 1,000 person-years. ^c^Adjusted for age and sex. ^d^Adjusted for age, sex, income, smoking status, regular physical activity, and CCI score. ^e^Adjusted for age, sex, income, smoking status, regular physical activity, type 2 diabetes mellitus, hypertension, dyslipidemia, chronic kidney disease, chronic heart disease, chronic lung disease, and immunocompromised status.

Abbreviations: ALD: alcohol-related liver disease; CCI: Charlson comorbidity index; CI: confidence interval; HR: hazard ratio; IR: incidence rate; MASLD: metabolic dysfunction-associated steatotic liver disease; MetALD: metabolic dysfunction and alcohol-related liver disease; Ref.: reference; SLD: steatotic liver disease.

In unadjusted analysis (Model 1), ALD (HR 2.05, 95% CI 1.98–2.13) and MASLD (HR 1.42, 95% CI 1.40–1.44) were associated with higher sepsis risk, whereas MetALD was not significantly associated with sepsis risk (HR 1.03, 95% CI 0.99–1.06). In the age- and sex-adjusted model (Model 2), all three SLD subtypes were significantly associated with increased sepsis risk, with HRs of 1.20 (95% CI 1.18–1.21) for MASLD, 1.26 (95% CI 1.22–1.31) for MetALD, and 1.84 (95% CI 1.77–1.91) for ALD (Table 2).

In multivariable-adjusted analysis (Model 3), which included age, sex, income, smoking status, regular physical activity, and CCI score, all three SLD subtypes remained significantly associated with increased sepsis risk. The highest risk was observed in the ALD group (adjusted HR [aHR] 1.52, 95% CI 1.47–1.58), followed by MetALD (aHR 1.17, 95% CI 1.13–1.21) and MASLD (aHR 1.13, 95% CI 1.12–1.15) (all *p* < 0.001). In Model 4, which adjusted for the same covariates as Model 3 but replaced the CCI score with individual comorbidities, chronic organ diseases, and immunocompromised status, risk estimates were slightly attenuated but remained statistically significant: ALD (aHR 1.53, 95% CI 1.48–1.59), MetALD (aHR 1.11, 95% CI 1.07–1.15), and MASLD (aHR 1.07, 95% CI 1.06–1.09) (all *p* < 0.001) (Table 2).

**Figure 2.**
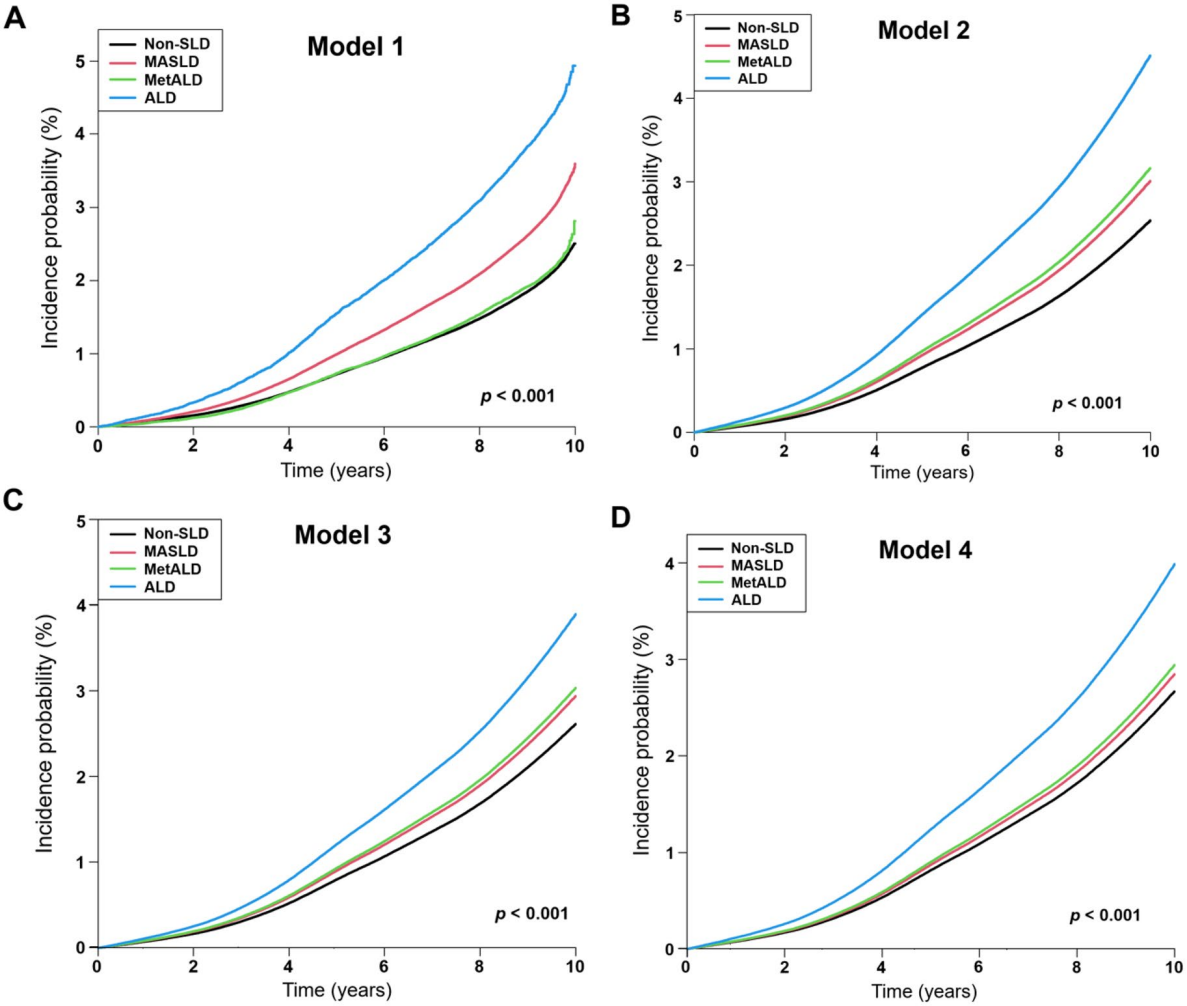
Kaplan–Meier curves for the cumulative incidence of sepsis by steatotic liver disease subtype. (A) Unadjusted model (Model 1), (B) adjusted for age and sex (Model 2), (C) adjusted for age, sex, income level, smoking status, regular physical activity, and CCI score (Model 3), and (D) adjusted for age, sex, income level, smoking status, regular physical activity, type 2 diabetes mellitus, hypertension, dyslipidemia, chronic kidney disease, chronic heart disease, chronic lung disease, and immunocompromised status (Model 4). All group comparisons were statistically significant (log-rank *p* < 0.001). Abbreviation: CCI: Charlson Comorbidity Index.

Kaplan–Meier curves demonstrated significant differences in cumulative sepsis incidence across SLD subtypes during follow-up (log-rank *p* < 0.001). Across all models, the ALD group consistently showed the highest cumulative incidence of sepsis. The relative ordering of risk across subtypes was similar in Models 3 and 4, indicating that replacing the CCI score with individual comorbidities, chronic organ diseases, and immunocompromised status did not materially change the overall pattern (Figure 2).

### Subgroup analyses

**Figure 3.**
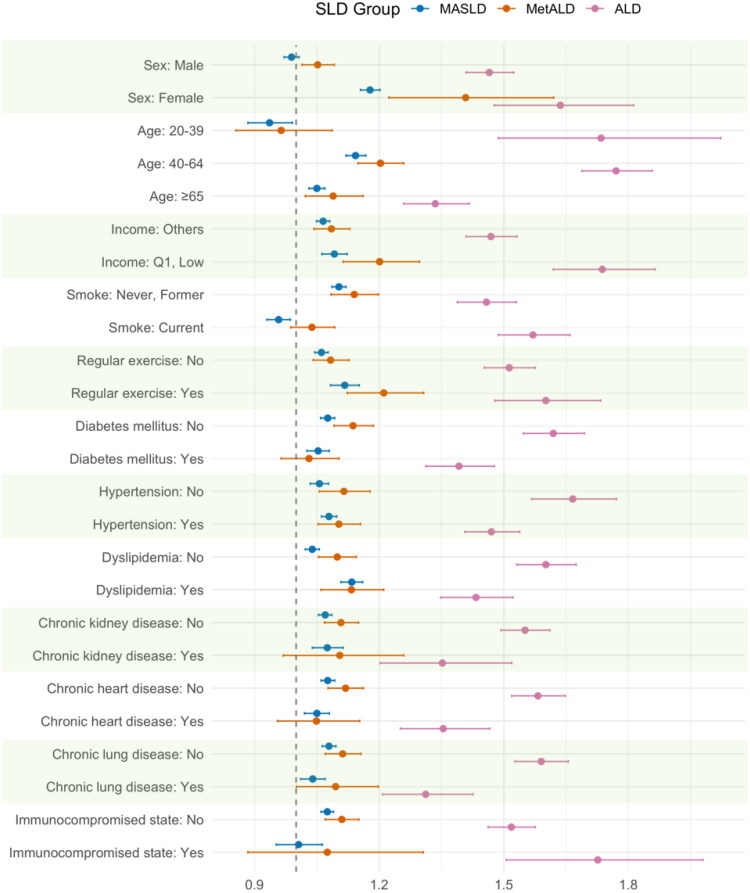
Stratified analyses of adjusted hazard ratios for sepsis according to SLD subtype. Forest plot showing adjusted hazard ratios (dots) and 95% confidence intervals (horizontal bars) for sepsis in MASLD, MetALD, and ALD, with the Non-SLD group as the reference. All estimates are from Model 4, which was adjusted for age, sex, income level, smoking status, regular physical activity, type 2 diabetes mellitus, hypertension, dyslipidemia, immunocompromised status, and chronic kidney, heart, and lung diseases. Abbreviations: ALD: alcohol-related liver disease; MASLD: metabolic dysfunction-associated steatotic liver disease; MetALD: metabolic dysfunction and alcohol-related liver disease; SLD: steatotic liver disease.

In subgroup analyses, the association between SLD subtypes and sepsis risk varied by sex, age group, and income level (p for interaction < 0.001). Within each SLD subtype, the relative risk of sepsis was generally higher in females than in males, most pronounced among individuals aged 40–64 years, and greater among those with low income (Table S7, Figure 3). Individuals in the lowest income group consistently showed elevated sepsis risk within each subtype, particularly in the ALD group (aHR 1.7, 95% CI 1.6–1.8 in Models 3 and 4).

The association between SLD subtype and sepsis risk differed by smoking status (p for interaction < 0.001). In stratified analyses, the relative risk estimates were lower among current smokers than among never or former smokers in the MASLD and MetALD groups, whereas current smoking was associated with higher sepsis risk in the ALD group (Table S7, Figure 3).

Across SLD subtypes, individuals who reported regular physical activity showed a higher relative risk of sepsis than those who did not report regular physical activity (Table S7, Figure 3). Across covariate-defined subgroups, including strata defined by individual risk factors and CCI score, the overall risk pattern was generally consistent, with the highest risk observed in ALD, followed by MetALD and MASLD (Table S7, Figure 3).

### Sensitivity analyses

We performed several sensitivity analyses to evaluate the robustness of the primary findings to alternative exposure definitions, outcome definitions, and potential baseline confounding. First, using FLI ≥60 as a more stringent threshold for SLD yielded findings comparable to those of the primary analysis. In the fully adjusted model, the aHRs were 1.201 (95% CI, 1.178–1.224) for MASLD, 1.250 (95% CI, 1.193–1.311) for MetALD, and 1.617 (95% CI, 1.538–1.700) for ALD (Table S8).

Second, we excluded 47,882 individuals with FLI <30 who nevertheless met the heavy alcohol intake criterion or had ICD-10 codes for ALD or alcohol abuse/misuse from the non-SLD reference group. The fully adjusted estimates were slightly higher than those in the primary analysis, with aHRs of 1.092 (95% CI, 1.077–1.107) for MASLD, 1.150 (95% CI, 1.110–1.191) for MetALD, and 1.568 (95% CI, 1.511–1.626) for ALD (Table S9).

Third, excluding ICD-10 code R65.0 from the outcome definition did not materially alter the findings. All three SLD subtypes remained significantly associated with increased sepsis risk, with fully adjusted aHRs of 1.079 (95% CI, 1.065–1.094) for MASLD, 1.133 (95% CI, 1.093–1.173) for MetALD, and 1.546 (95% CI, 1.490–1.604) for ALD (Table S10).

Fourth, in the Fine–Gray competing-risk analysis treating death before hospitalization for sepsis as a competing event, the findings remained consistent with the primary analysis. In the fully adjusted model, the sHRs were 1.119 (95% CI, 1.104–1.134) for MASLD, 1.167 (95% CI, 1.127–1.209) for MetALD, and 1.569 (95% CI, 1.512–1.628) for ALD (Table S11).

Fifth, when follow-up was restricted to 5 years, all three SLD subtypes remained significantly associated with increased sepsis risk, with fully adjusted aHRs of 1.046 (95% CI, 1.023–1.070) for MASLD, 1.162 (95% CI, 1.095–1.232) for MetALD, and 1.579 (95% CI, 1.486–1.677) for ALD. In the 3-year analysis, ALD remained significantly associated with increased sepsis risk (aHR 1.709, 95% CI, 1.560–1.874), whereas the estimates for MASLD (aHR 1.015, 95% CI, 0.979–1.053) and MetALD (aHR 1.064, 95% CI, 0.965–1.173) were attenuated and not statistically significant (Table S12).

Finally, to minimize potential confounding by baseline comorbidities, we conducted a sensitivity analysis restricted to individuals with a CCI score of 0 (*n* = 2,194,543; Table 3). The direction of the associations between SLD subtypes and sepsis risk was consistent with that observed in the primary analysis. In the fully adjusted model (Model 4), MASLD (aHR 1.09, 95% CI 1.06–1.12), MetALD (aHR 1.16, 95% CI 1.09–1.24), and ALD (aHR 1.60, 95% CI 1.47–1.76) each remained significantly associated with increased risk of sepsis compared with the non-SLD group (all *p* < 0.001). Across all sensitivity analyses, the overall risk gradient remained unchanged, with the highest risk observed in ALD, followed by MetALD and MASLD.

**Table 3.** Sensitivity analysis of sepsis risk across SLD subtypes among individuals with a charlson comorbidity index score of 0.

SLD subtypes	Total individuals	Sepsis^a^	Duration of follow-up (years)	IR^b^	HR (95% CI)			
					Unadjusted model 1	Adjusted model 2^c^	Adjusted model 3^d^	Adjusted model 4^e^
Non-SLD	1,460,886	15,330	13,557,751	1.13	1 (Ref.)	1 (Ref.)	1 (Ref.)	1 (Ref.)
MASLD	620,443	8,495	5,729,607	1.48	1.318 (1.283–1.353)	1.147 (1.116–1.179)	1.135 (1.104–1.167)	1.086 (1.056–1.117)
MetALD	88,505	1,130	816,592	1.38	1.231 (1.159–1.308)	1.296 (1.218–1.378)	1.231 (1.157–1.310)	1.161 (1.091–1.236)
ALD	24,709	502	227,052	2.21	1.958 (1.791–2.140)	1.798 (1.644–1.967)	1.711 (1.564–1.872)	1.603 (1.465–1.755)

^a^Refers to the first episode of sepsis requiring hospitalization. ^b^Per 1,000 person-years. ^c^Adjusted for age and sex. ^d^Adjusted for age, sex, income, smoking status, regular physical activity, and CCI score. ^e^Adjusted for age, sex, income, smoking status, regular physical activity, type 2 diabetes mellitus, hypertension, dyslipidemia, chronic kidney disease, chronic heart disease, chronic lung disease, and immunocompromised status.

**Abbreviations:** ALD: alcohol-related liver disease; CCI: Charlson comorbidity index; CI: confidence interval; HR: hazard ratio; IR: incidence rate; MASLD: metabolic dysfunction-associated steatotic liver disease; MetALD: metabolic dysfunction and alcohol-related liver disease; Ref.: reference; SLD: steatotic liver disease.

## Discussion

### Principal findings

In this large-scale, nationwide cohort study, we found that the newly defined SLD subtypes – MASLD, MetALD, and ALD – were each associated with an increased risk of hospitalization for sepsis. The magnitude of risk differed across subtypes, with ALD showing the highest risk, followed by MetALD and MASLD. These associations remained significant after adjustment for demographic characteristics, lifestyle factors, and comorbid conditions, and were consistent in sensitivity analyses restricted to individuals with a CCI score of 0. These findings suggest that the association between SLD subtypes and sepsis risk is not fully explained by measured baseline comorbidity burden. Particularly, the higher risk observed in ALD highlights the potential importance of alcohol-related hepatic injury in susceptibility to severe infection.

### Comparison with previous studies and potential mechanisms

Our findings extend previous studies reporting increased infectious complications and mortality in patients with chronic liver disease, particularly ALD [22,23]. Chronic hepatic steatosis and alcohol-related liver injury may impair innate and adaptive immune responses through mechanisms such as reduced neutrophil chemotaxis, altered Kupffer cell function, dysregulated cytokine signaling, and impaired host defense [24,25]. In ALD, chronic ethanol exposure can promote gut barrier dysfunction and endotoxemia, leading to immune dysregulation characterized by impaired neutrophil function, altered antigen presentation, dysregulated cytokine responses, and increased intestinal permeability, which may collectively facilitate bacterial translocation, systemic inflammation, and susceptibility to severe infections [22,26–29]. Li et al. demonstrated that advanced ALD is associated with reduced antigen-presenting capacity of innate immune cells and dysfunctional T-cell responses [25]. The substantially higher risk observed in ALD in our cohort is consistent with these biological mechanisms and supports the biological plausibility of the observed association.

In contrast, the more modest but significant increase in sepsis risk observed in MASLD and MetALD may reflect chronic low-grade inflammation, metabolic endotoxemia, gut–liver axis dysfunction, and immune-metabolic derangements associated with metabolic liver disease [11,30,31]. MetALD represents a clinically important overlap state in which metabolic dysfunction and increased alcohol intake coexist [32]. In the crude analysis, sepsis risk in the MetALD group was similar to that in the non-SLD group; however, the risk estimate increased after adjustment for age and sex, suggesting that differences in demographic composition attenuated the unadjusted association. Individuals with MetALD were younger and had fewer comorbidities than those with MASLD or ALD, yet their fully adjusted sepsis risk exceeded that of MASLD. This pattern is compatible with an additional contribution of alcohol intake to infection susceptibility in the presence of metabolic dysfunction. This interpretation is supported by population-based evidence showing a J-shaped relationship between alcohol intake and infection risk, with heavier alcohol consumption associated with increased risks of serious infection and infection-related mortality [33]. These mechanisms support biological plausibility; however, the observational design cannot establish causality or exclude bidirectional relationships.

Subgroup analyses showed that the association between SLD subtypes and sepsis risk varied by sex, age, income level, smoking status, and physical activity. Females, individuals aged 40–64 years, and those with low income showed relatively higher sepsis risks within several SLD subtypes, particularly ALD. These findings may reflect differences in biological susceptibility, healthcare access, health behaviors, or residual confounding. Previous studies have reported sex differences, age-related vulnerability, and socioeconomic gradients in ALD and infection-related outcomes [34–36]. Regular physical activity was unexpectedly associated with higher sepsis risk across SLD subtypes. This counterintuitive finding may reflect reverse causation, residual confounding, or differences in health-seeking behavior rather than a harmful effect of exercise itself [37]. Smoking showed divergent patterns, with lower relative risk in MASLD and MetALD but higher risk in ALD. The lower risk observed among current smokers in some subgroups should be interpreted cautiously, as it may be influenced by unmeasured confounding, competing risks, or selection effects. In contrast, the higher risk among current smokers with ALD may be biologically plausible, given prior evidence that concurrent alcohol and tobacco exposure may impair immune responses [38,39]. Overall, these observational subgroup findings should not be interpreted as evidence of causal effects of exercise or smoking.

### Clinical implications

These findings have potential implications for risk stratification and preventive care in individuals with SLD. The observed risk hierarchy – ALD showing the highest adjusted risk, followed by MetALD and MASLD – suggests that SLD subtype may help identify individuals who warrant closer attention for infection-related complications. Patients with ALD may represent a particularly high-risk group in whom vaccination, early recognition of infection, alcohol cessation support, and timely medical evaluation during acute illness should be emphasized. Individuals with MetALD may benefit from both metabolic risk management and alcohol reduction strategies, whereas those with MASLD may require infection awareness and optimization of cardiometabolic comorbidities.

The subgroup findings may further help identify populations at higher relative risk, including individuals with ALD who are female, middle-aged, or socioeconomically disadvantaged. However, because these analyses involved multiple comparisons and may be susceptible to residual confounding, the findings should be interpreted as exploratory and hypothesis-generating rather than confirmatory and should be validated in future studies. If validated, SLD subtype and selected clinical characteristics could be incorporated into clinical risk assessment tools or population health strategies to support targeted infection-prevention efforts.

### Strengths and limitations

This study has several strengths. First, we evaluated sepsis risk according to the newly defined SLD subtypes in a large, nationwide population-based cohort with long-term follow-up. Second, the linkage of health screening data with claims records enabled assessment of anthropometric measures, laboratory findings, lifestyle factors, comorbidities, immunocompromised status, and longitudinal hospitalization outcomes. Third, the associations were evaluated using sequentially adjusted models and sensitivity analyses restricted to individuals without baseline comorbidity burden as measured by CCI.

Several limitations should be acknowledged. First, SLD was defined using FLI rather than imaging- or biopsy-based assessment of hepatic steatosis. Although FLI does not capture fibrosis stage, steatohepatitis, or other markers of liver disease severity, the primary aim of this study was to compare sepsis risk across etiologic SLD subtypes rather than to assess liver disease severity. In this context, FLI provided a practical and standardized approach for classifying SLD in this large-scale health screening cohort. FLI has been validated in community-based populations and has shown acceptable performance for detecting ultrasound-defined fatty liver [14,40,41]. Recent studies have also used FLI to define MASLD in large-scale population-based settings [42–44], supporting its feasibility for epidemiologic research. Nevertheless, because FLI incorporates body mass index and waist circumference, individuals with advanced alcohol-related liver disease accompanied by sarcopenia or low adiposity may have had FLI values <30 and been classified as non-SLD. In a sensitivity analysis excluding individuals with FLI <30 who met the heavy alcohol intake criterion or had relevant ICD-10 codes from the non-SLD reference group, the associations remained consistent and were slightly stronger than those in the primary analysis. These findings suggest that this potential misclassification did not materially affect the main results.

Second, sepsis was identified using ICD-10 codes from hospitalization claims. Claims-based definitions may underestimate the true incidence of sepsis because of limited sensitivity [45,46]. To reduce outcome misclassification and capture clinically meaningful events, we restricted the outcome to sepsis requiring hospitalization and excluded outpatient-only diagnoses. Previous evidence suggests that ICD-10 coding for sepsis has relatively high specificity [46], indicating a low likelihood of false-positive outcome classification. Because all SLD subtypes were assessed using the same outcome definition, any under-ascertainment would be expected to affect all groups similarly, thereby reducing the likelihood that relative risk comparisons were materially distorted.

Third, SLD subtype was classified using information obtained at baseline and was treated as fixed throughout follow-up. Alcohol intake, cardiometabolic status, body weight, and liver disease status may have changed over time, potentially resulting in time-dependent exposure misclassification. In sensitivity analyses restricted to shorter follow-up periods, ALD remained significantly associated with increased sepsis risk at both 3 and 5 years, whereas the associations for MASLD and MetALD were not statistically significant in the 3-year analysis but were statistically significant in the 5-year analysis. The findings for MASLD and MetALD at 3 years may partly reflect fewer accumulated sepsis events and lower precision during the shorter follow-up period.

Fourth, alcohol intake, smoking, and physical activity were self-reported at baseline and may be subject to measurement error or underreporting. Nevertheless, alcohol intake was collected using a standardized health screening questionnaire and classified using predefined sex-specific thresholds consistent with international SLD nomenclature. In addition, the alcohol-based subtype definitions produced expected baseline patterns, including moderate alcohol consumption in MetALD and heavy alcohol consumption in a substantial proportion of ALD, supporting the internal consistency of the classification. Residual confounding by unmeasured factors, including dietary patterns and differences in healthcare utilization, cannot be excluded despite multivariable adjustment and sensitivity analyses. Therefore, the observed associations should not be interpreted as establishing causality. In addition, because this study was conducted exclusively in a Korean population, the generalizability of the findings may be limited by population-level differences in body composition and FLI performance, alcohol metabolism and drinking patterns, diagnostic coding practices, and infectious disease epidemiology. Validation in ethnically and geographically diverse populations is therefore warranted.

Despite these limitations, the consistent association between SLD subtype and sepsis risk across multiple models and sensitivity analyses supports the robustness of the observed associations.

## Conclusion

In conclusion, this nationwide cohort study showed that MASLD, MetALD, and ALD were each associated with an increased risk of hospitalization for sepsis, with the highest risk observed in ALD. These findings suggest that the newly defined SLD subtypes may help identify individuals at increased risk of severe infection beyond conventional comorbidity-based assessment. Subtype-specific risk assessment, particularly recognition of the elevated risk associated with ALD and MetALD, may help inform infection-prevention strategies and clinical surveillance in patients with steatotic liver disease.

## Supplementary Material

Supplementary Table 7_Final.xlsx

Supplementary materials_revised_260706.docx

## Data Availability

The data underlying this article cannot be shared publicly because they are derived from health insurance claims and health screening records subject to ethical, privacy, and legal restrictions imposed by the Korean National Health Insurance Service (NHIS). The data may be made available upon reasonable request through the NHIS Big Data Platform (https://nhiss.nhis.or.kr/) and after approval of a research proposal by the NHIS. Researchers who meet the criteria for access to confidential data may apply through the NHIS Data Request system (https://nhiss.nhis.or.kr/lp/a/b/100/lpab100m01.do). The authors are not permitted to share the dataset directly. This study was conducted using fully anonymized data provided by the NHIS under application number REQ2025040336-001.
