# Supplementary material for: Steatotic liver disease subtypes and risk of hospitalization for sepsis: a nationwide cohort study
Source: Ann Med. 2026 Jul 25;58(1):2704290. doi: 10.1080/07853890.2026.2704290 (PMC13403459; doi:10.1080/07853890.2026.2704290)
Supplement: Supplementary materials_revised_260706.docx [file IANN_A_2704290_SM7367.docx]

**Table S1.** **Self-reported medical history, lifestyle factors, and health-related behaviors collected through the Korean National Health Screening Program**

| **Questions related to disease history** (past history and family history)  Please read the questions below and mark '√' the information that corresponds to your current status.  **1**. Have you been diagnosed with any of the following diseases or are you currently  receiving medication?   \|  \| Stroke \| CVD  (Angina/MI) \| HTN \| T2DM \| Dyslipidemia \| Others  (Includes cancer) \| \| --- \| --- \| --- \| --- \| --- \| --- \| --- \| \| Diagnosis \|  \|  \|  \|  \|  \|  \| \| Medication status \|  \|  \|  \|  \|  \|  \|   CVD: cardiovascular disease, MI: Myocardial infarction, HTN: hypertension,  T2DM: type 2 diabetes mellitus  **2**. Have any of your parents, brothers, or sisters suffered from or died from the following  diseases?   \|  \| Stroke \| CVD  (Angina/MI) \| HTN \| T2DM \| Dyslipidemia \| Others  (Includes cancer) \| \| --- \| --- \| --- \| --- \| --- \| --- \| --- \| \| Yes \|  \|  \|  \|  \|  \|  \|   **3**. Are you a hepatitis B antigen carrier?  ① Yes ② No ③ Don't know | | | | | | | | | |
| --- | --- | --- | --- | --- | --- | --- | --- | --- | --- | --- | --- | --- | --- | --- | --- | --- | --- | --- | --- | --- | --- | --- | --- | --- | --- | --- | --- | --- | --- | --- | --- | --- | --- | --- | --- | --- | --- | --- | --- | --- | --- | --- | --- | --- |
| **Physical activities**  Please read the following items and mark '√' on the response that corresponds to your activity status during the past week. | | | | | | | | | |
| **1**. How many days in the past week did you engage in intense activities that made you breathe much harder than usual for more than 20 minutes? (e.g., running, aerobics, fast cycling, hiking, etc.) | | | | | | | | | |
| 0 | 1 | 2 | | 3 | 4 | 5 | | 6 | 7 |
| **2**. How many days in the past week did you engage in moderate activities that made you breathe slightly harder than usual for more than 30 minutes? (e.g., brisk walking, playing tennis, cycling at a regular pace, mopping, etc.) Exclude activities related to the response in 1. | | | | | | | | | |
| 0 | 1 | 2 | | 3 | 4 | 5 | | 6 | 7 |
| **3**. How many days in the past week did you walk for at least 30 minutes, combining walks of at least 10 minutes each time? (e.g., walking during commuting or leisure time) Exclude physical activities related to the responses in 1 and 2. | | | | | | | | | |
| 0 | 1 | 2 | | 3 | 4 | 5 | | 6 | 7 |
| **Smoking habits** | | | | | | | | | |
| Please read the following items and provide information that corresponds to your current condition. | | | | | | | | | |
| **1**. Have you smoked a total of 5 packs (100 cigarettes) or more in your lifetime? | | | | | | | | | |
| (1) No | | | (2) Yes, but I quit.  → Go to questionnaire 2 | | | | (3) Yes, I am smoking.  → Go to questionnaire 3 | | |
| **2**. If you are an ex-smoker, | | | | | | | | | |
| \| How many years did you smoke? \| Total ______ years \| \| --- \| --- \| \| How many cigarettes per day? \| ______ cigarettes \| | | | | | | | | | |
| **3**. If you are a current smoker, | | | | | | | | | |
| \| How many years have you been smoking? \| Total ______ years \| \| --- \| --- \| \| How many cigarettes per day? \| ______ cigarettes \| | | | | | | | | | |
| **Drinking habits** | | | | | | | | | |
| Please read the following items and provide information that corresponds to your current condition. | | | | | | | | | |
| **1**. On average, how many days a week do you drink alcohol? | | | | | | | | | |
| 0 | 1 | 2 | | 3 | 4 | 5 | | 6 | 7 |
| **2**. How much do you usually drink per day (regardless of the type of alcohol)? | | | | | | | | | |
| ______ glasses | | | | | | | | | |

**Table S2. ICD-10 codes used to define chronic liver diseases and alcohol-related conditions**

| **Category** | **Diagnosis** | **ICD-10 Code** |
| --- | --- | --- |
| **Alcohol abuse/misuse** | Alcohol abuse, dependence, or intoxication | E24.4, F10, G31.2, G62.1, G72.1, I42.6, K29.2, K86.0, Q35.4, R78.0, T51.0, T51.8, T51.9, X65, Y15, Y57.3, Y90, Y91, Z50.2, Z71.4, Z71.2 |
|  | Other abuse- and drug-related diagnoses | F11–F19 |
| **Alcohol-related liver disease** | Alcoholic liver disease | K70 |
|  | Alcoholic fatty liver | K70.0 |
|  | Alcoholic hepatitis | K70.1 |
|  | Alcoholic fibrosis and sclerosis  of liver | K70.2 |
|  | Alcoholic cirrhosis of liver | K70.3 |
|  | Alcoholic hepatic failure | K70.4 |
|  | Alcoholic liver disease,  unspecified | K70.9 |
| **Chronic liver diseases** | Drug-induced liver disease | K71 |
|  | Viral hepatitis  (e.g., hepatitis B, C) | B15–B19, B00.8, B25.1 |
|  | Hepatic veno-occlusive disease (Budd-Chiari syndrome) | I82 |
|  | Liver abscess | K75.0, A06.4 |
|  | Hemochromatosis | E83.1 |
|  | Wilson’s disease | E83.0 |
|  | Alpha-1 antitrypsin deficiency | E88.0 |
|  | Autoimmune hepatitis | K75.4 |
|  | Primary biliary cholangitis | K74.3, K74.4 |
|  | Other cholangitis | K83 |
|  | Glycogen storage disease | E74 |

Abbreviations: ICD-10, International Classification of Diseases and Related Health Problems, 10th Revision, Tenth Revision (2019).

**Table S3.** List of diagnosis codes used to extract patients with sepsis

| **ICD-10 code** | **Diagnosis** |
| --- | --- |
| **A02.1** | Salmonella sepsis |
| **A20.7** | Septicemic plague |
| **A22.7** | Anthrax sepsis |
| **A26.7** | Erysipelothrix sepsis |
| **A32.7** | Listerial sepsis |
| **A40** | ***Streptococcal* sepsis** |
| A40.0 | Sepsis due to *Streptococcus*, group A |
| A40.1 | Sepsis due to *Streptococcus*, group B |
| A40.2 | Sepsis due to *Streptococcus*, group D and *enterococcus* |
| A40.3 | Sepsis due to *Streptococcus pneumoniae* (Pneumococcal sepsis) |
| A40.8 | Other *Streptococcal* sepsis |
| A40.9 | *Streptococcal* sepsis, unspecified |
| **A41** | **Other sepsis** |
| A41.0 | Sepsis due to *Staphylococcus aureus* |
| A41.1 | Sepsis due to other specified *Staphylococcus*  (Sepsis due to coagulase-negative *Staphylococcus*) |
| A41.2 | Sepsis due to unspecified *Staphylococcus* |
| A41.3 | Sepsis due to *Haemophilus influenzae* |
| A41.4 | Sepsis due to anaerobes |
| A41.5 | Sepsis due to other Gram-negative organisms  (Gram-negative sepsis NOS) |
| A41.8 | Other specified sepsis |
| A41.9 | Sepsis, unspecified (Septicemia) |
| **A42.7** | Actinomycotic sepsis |
| **B37.7** | Candidal sepsis |
| **R57.2** | Septic shock |
| **R65** | **Systemic Inflammatory Response Syndrome** |
| R65.0 | Systemic Inflammatory Response Syndrome of infectious origin without organ failure |
| R65.1 | Systemic Inflammatory Response Syndrome of infectious origin with organ failure |

No patients were identified with ICD-10 codes A20.7 or A22.7.

Abbreviations: NOS, not otherwise specified.

**Table S4. Definitions and classification of clinical variables**

| **Variables** | **Definitions** | | |
| --- | --- | --- | --- |
| **T2DM** | Patients taking anti-diabetic drugs with E11-14  or fasting glucose ≥126 mg/dL | | |
| **Hypertension** | Patients taking anti-hypertensive drugs with I10-I13, I15  or SBP ≥140 mmHg, or DBP ≥90 mmHg | | |
| **Dyslipidemia** | Patients taking dyslipidemia agents with E78  or fasting total cholesterol ≥240 mg/dL | | |
| **CKD** | eGFR <60 mL/min/1.73 m² by MDRD equation  or a history of ESRD identified by special registration codes in the Korean NHIS (V001 - HD, V003 - PD, V005 - post-kidney transplantation care involving immunosuppressive therapy | | |
| **BMI categories** | Underweight | < 18.5 kg/m^2^ | |
|  | Normal | 18.5-22.9 kg/m^2^ | |
|  | Overweight at risk (pre-obese) | 23.0-24.9 kg/m^2^ | |
|  | Obese class I | 25.0-29.9 kg/m^2^ | |
|  | Obese class II | ≥ 30.0 kg/m^2^ | |
| **Abdominal obesity** | Waist circumference ≥ 90 cm in male and ≥ 85 cm in female | | |
| **Alcohol consumption**  **(g/day)** |  | Men | Women |
|  | None (abstainers) | 0 | 0 |
|  | Low drinkers | <30 | <20 |
|  | Moderate drinkers | 30-60 | 20-50 |
|  | Heavy drinkers | ≥ 60 | ≥ 50 |

The diagnostic codes were applied based on the ICD-10 classification. The GFR was calculated using the MDRD equation: 186.3 × (serum creatinine)^−1.154^ × (age)^−0.203^, applying a factor of 0.742 for females and 1.0 for males.

Abbreviations: BMI, body mass index; CKD, chronic kidney diseases; DBP, diastolic blood pressure; eGFR, estimated glomerular filtration rate; ESRD, end-stage renal disease; HD, hemodialysis; MDRD, modification of diet in renal disease; NHIS, National Health Insurance Service; PD, peritoneal dialysis; SBP, systolic blood pressure; T2DM, type 2 diabetes mellitus.

**Table S5.** **ICD-10 codes used to define the components of the Charlson Comorbidity Index score**

| **Comorbidities** | **ICD-10 codes** | **Original weight** |
| --- | --- | --- |
| **Myocardial infarction** | I21.x, I22.x, I25.2 | 1 |
| **Congestive heart failure** | I09.9, I11.0, I13.0, I13.2, I25.5, I42.0, I42.5–I42.9, I43.x, I50.x, P29.0 | 1 |
| **Peripheral vascular disease** | I70.x, I71.x, I73.1, I73.8, I73.9, I77.1, I79.0, I79.2, K55.1, K55.8, K55.9, Z95.8, Z95.9 | 1 |
| **Cerebrovascular disease** | G45.x, G46.x, H34.0, I60.x–I69.x | 1 |
| **Dementia** | F00.x–F03.x, F05.1, G30.x, G31.1 | 1 |
| **Chronic pulmonary disease** | I27.8, I27.9, J40.x–J47.x, J60.x–J67.x, J68.4, J70.1, J70.3 | 1 |
| **Rheumatologic disease** | M05.x, M06.x, M31.5, M32.x–M34.x, M35.1, M35.3, M36.0 | 1 |
| **Peptic ulcer disease** | K25.x–K28.x | 1 |
| **Mild liver disease** | B18.x, K70.0–K70.3, K70.9, K71.3–K71.5, K71.7, K73.x, K74.x, K76.0, K76.2–K76.4, K76.8, K76.9, Z94.4 | 1 |
| **Diabetes without chronic complication** | E10.0, E10.1, E10.6, E10.8, E10.9, E11.0, E11.1, E11.6, E11.8, E11.9, E12.0, E12.1, E12.6, E12.8, E12.9, E13.0, E13.1, E13.6, E13.8, E13.9, E14.0, E14.1, E14.6, E14.8, E14.9 | 1 |
| **Diabetes with chronic complication** | E10.2–E10.5, E10.7, E11.2–E11.5, E11.7, E12.2–E12.5, E12.7, E13.2–E13.5, E13.7, E14.2–E14.5, E14.7 | 2 |
| **Hemiplegia or paraplegia** | G04.1, G11.4, G80.1, G80.2, G81.x, G82.x, G83.0–G83.4, G83.9 | 2 |
| **Renal disease** | I12.0, I13.1, N03.2–N03.7, N05.2–N05.7, N18.x, N19.x, N25.0, Z49.0–Z49.2, Z94.0, Z99.2 | 2 |
| **Any malignancy including**  **leukemia and lymphoma** | C00.x–C26.x, C30.x–C34.x, C37.x–C41.x, C43.x, C45.x–C58.x, C60.x–C76.x, C81.x–C85.x, C88.x, C90.x–C97.x | 2 |
| **Moderate or severe liver disease** | I85.0, I85.9, I86.4, I98.2, K70.4, K71.1, K72.1, K72.9, K76.5, K76.6, K76.7 | 3 |
| **Metastatic solid tumor** | C77.x–C80.x | 6 |
| **Acquired immune deficiency syndrome/HIV** | B20.x–B22.x, B24.x | 6 |

**Table S6. ICD-10 classification of established risk factors for sepsis**

| Disease category | Code |
| --- | --- |
| Immunocompromised state |  |
| Solid organ transplant | Z94.0-Z94.3, Z94.8, Z94.9 (Exclude liver Tx) |
| Hematologic malignancy | C81-96 |
| Solid cancer | C00-80, C97 |
| HIV infection and hyposplenism | B20-24, D73.0 |
| Comorbidities |  |
| Chronic heart disease | I11, I13.0, I13.2, I13.9, I20-25, I27, I34-37, I39.0-39.4, I42-43, I44-I45, I47-I49, I50 |
| Chronic lung disease | J42-J47, J60-65, J84, J98.2, J98.3 |

Abbreviation: HIV, human immunodeficiency virus; Tx, transplantation

**Table S8. Sensitivity analysis of the risk of hospitalization for sepsis across steatotic liver disease subtypes using a fatty liver index ≥60 as the operational threshold for steatotic liver disease**

| **SLD subtypes** | **HR (95% CI)** | | | |
| --- | --- | --- | --- | --- |
|  | **Unadjusted**  **Model 1** | **Adjusted**  **model 2**^a^ | **Adjusted**  **model 3**^b^ | **Adjusted**  **model 4**^c^ |
| **Non-SLD** | 1 (Ref.) | 1 (Ref.) | 1 (Ref.) | 1 (Ref.) |
| **MASLD** | 1.261(1.238-1.285) | 1.350(1.325-1.376) | 1.272(1.248-1.296) | 1.201(1.178-1.224) |
| **MetALD** | 1.019(0.973-1.067) | 1.414(1.349-1.481) | 1.335(1.274-1.398) | 1.250(1.193-1.311) |
| **ALD** | 1.786(1.700-1.877) | 1.890(1.798-1.986) | 1.567(1.491-1.648) | 1.617(1.538-1.700) |

^a^Adjusted for age and sex. ^b^Adjusted for age, sex, income, smoking status, regular physical activity, and CCI score. ^c^Adjusted for age, sex, income, smoking status, regular physical activity, type 2 diabetes mellitus, hypertension, dyslipidemia, chronic kidney disease, chronic heart disease, chronic lung disease, and immunocompromised status.

Abbreviations, ALD, alcohol-related liver disease; CCI, Charlson comorbidity index; CI, confidence interval; HR, hazard ratio; IR, incidence rate; MASLD, metabolic dysfunction-associated steatotic liver disease; MetALD, metabolic dysfunction and alcohol-related liver disease; Ref., reference, SLD, steatotic liver disease.

**Table S9. Sensitivity analysis of the risk of hospitalization for sepsis after excluding individuals with a fatty liver index <30 who met the heavy alcohol intake criterion or had ICD-10 codes for alcohol-related liver disease or alcohol abuse/misuse from the non-SLD reference group**

| **SLD subtypes** | **HR (95% CI)** | | | |
| --- | --- | --- | --- | --- |
|  | **Unadjusted**  **Model 1** | **Adjusted**  **model 2**^a^ | **Adjusted**  **model 3**^b^ | **Adjusted**  **model 4**^c^ |
| **Non-SLD** | 1(Ref.) | 1(Ref.) | 1(Ref.) | 1(Ref.) |
| **MASLD** | 1.440(1.421-1.458) | 1.198(1.182-1.214) | 1.136(1.121-1.151) | 1.092(1.077-1.107) |
| **MetALD** | 1.064(1.028-1.101) | 1.292(1.247-1.338) | 1.213(1.171-1.256) | 1.150(1.110-1.191) |
| **ALD** | 2.059(1.986-2.134) | 1.820(1.755-1.887) | 1.499(1.445-1.555) | 1.568(1.511-1.626) |

^a^Adjusted for age and sex. ^b^Adjusted for age, sex, income, smoking status, regular physical activity, and CCI score. ^c^Adjusted for age, sex, income, smoking status, regular physical activity, type 2 diabetes mellitus, hypertension, dyslipidemia, chronic kidney disease, chronic heart disease, chronic lung disease, and immunocompromised status.

Abbreviations, ALD, alcohol-related liver disease; CCI, Charlson comorbidity index; CI, confidence interval; HR, hazard ratio; IR, incidence rate; MASLD, metabolic dysfunction-associated steatotic liver disease; MetALD, metabolic dysfunction and alcohol-related liver disease; Ref., reference, SLD, steatotic liver disease.

**Table S10. Sensitivity analysis of the association between steatotic liver disease subtypes and sepsis risk after excluding ICD-10 code R65.0 from the outcome definition**

| **SLD subtypes** | **HR (95% CI)** | | | |
| --- | --- | --- | --- | --- |
|  | **Unadjusted**  **Model 1** | **Adjusted**  **model 2**^a^ | **Adjusted**  **model 3**^b^ | **Adjusted**  **model 4**^c^ |
| **Non-SLD** | 1(Ref.) | 1(Ref.) | 1(Ref.) | 1(Ref.) |
| **MASLD** | 1.419(1.401-1.438) | 1.182(1.167-1.198) | 1.126(1.111-1.141) | 1.079(1.065-1.094) |
| **MetALD** | 1.051(1.015-1.087) | 1.272(1.229-1.317) | 1.199(1.157-1.241) | 1.133(1.093-1.173) |
| **ALD** | 2.034(1.962-2.108) | 1.794(1.730-1.860) | 1.482(1.429-1.538) | 1.546(1.490-1.604) |

^a^Adjusted for age and sex. ^b^Adjusted for age, sex, income, smoking status, regular physical activity, and CCI score. ^c^Adjusted for age, sex, income, smoking status, regular physical activity, type 2 diabetes mellitus, hypertension, dyslipidemia, chronic kidney disease, chronic heart disease, chronic lung disease, and immunocompromised status.

Abbreviations, ALD, alcohol-related liver disease; CCI, Charlson comorbidity index; CI, confidence interval; HR, hazard ratio; IR, incidence rate; MASLD, metabolic dysfunction-associated steatotic liver disease; MetALD, metabolic dysfunction and alcohol-related liver disease; Ref., reference, SLD, steatotic liver disease.

**Table S11. Fine–Gray competing-risk analysis of the association between steatotic liver disease subtypes and sepsis risk, treating death before hospitalization for sepsis as a competing event**

| **SLD subtypes** | **sHR (95% CI)** | | | |
| --- | --- | --- | --- | --- |
|  | **Unadjusted**  **Model 1** | **Adjusted**  **model 2**^a^ | **Adjusted**  **model 3**^b^ | **Adjusted**  **model 4**^c^ |
| **Non-SLD** | 1(Ref.) | 1(Ref.) | 1(Ref.) | 1(Ref.) |
| **MASLD** | 1.413(1.395-1.431) | 1.213(1.197-1.229) | 1.162(1.147-1.178) | 1.119(1.104-1.134) |
| **MetALD** | 1.047(1.012-1.084) | 1.293(1.249-1.339) | 1.231(1.189-1.276) | 1.167(1.127-1.209) |
| **ALD** | 1.983(1.913-2.056) | 1.795(1.731-1.862) | 1.508(1.454-1.565) | 1.569(1.512-1.628) |

^a^Adjusted for age and sex. ^b^Adjusted for age, sex, income, smoking status, regular physical activity, and CCI score. ^c^Adjusted for age, sex, income, smoking status, regular physical activity, type 2 diabetes mellitus, hypertension, dyslipidemia, chronic kidney disease, chronic heart disease, chronic lung disease, and immunocompromised status.

Abbreviations, sHR, subdistribution hazard ratio; ALD, alcohol-related liver disease; CCI, Charlson comorbidity index; CI, confidence interval; HR, hazard ratio; IR, incidence rate; MASLD, metabolic dysfunction-associated steatotic liver disease; MetALD, metabolic dysfunction and alcohol-related liver disease; Ref., reference, SLD, steatotic liver disease.

**Table S12. Sensitivity analyses of the association between steatotic liver disease subtypes and sepsis risk restricted to 3- and 5-year follow-up**

**(a) 3-year follow-up**

| **SLD subtypes** | **HR (95% CI)** | | | |
| --- | --- | --- | --- | --- |
|  | **Unadjusted**  **Model 1** | **Adjusted**  **model 2**^a^ | **Adjusted**  **model 3**^b^ | **Adjusted**  **model 4**^c^ |
| **Non-SLD** | 1(Ref.) | 1(Ref.) | 1(Ref.) | 1(Ref.) |
| **MASLD** | 1.333(1.287-1.380) | 1.122(1.083-1.162) | 1.057(1.02-1.095) | 1.015(0.979-1.053) |
| **MetALD** | 0.938(0.853-1.032) | 1.181(1.072-1.300) | 1.127(1.023-1.242) | 1.064(0.965-1.173) |
| **ALD** | 2.239(2.047-2.449) | 1.982(1.81-2.171) | 1.58(1.442-1.732) | 1.709(1.56-1.874) |

**(b) 5-year follow-up**

| **SLD subtypes** | **HR (95% CI)** | | | |
| --- | --- | --- | --- | --- |
|  | **Unadjusted**  **Model 1** | **Adjusted**  **model 2**^a^ | **Adjusted**  **model 3**^b^ | **Adjusted**  **model 4**^c^ |
| **Non-SLD** | 1(Ref.) | 1(Ref.) | 1(Ref.) | 1(Ref.) |
| **MASLD** | 1.375(1.345-1.405) | 1.154(1.120-1.180) | 1.091(1.067-1.115) | 1.046(1.023-1.070) |
| **MetALD** | 1.056(0.997-1.118) | 1.299(1.225-1.376) | 1.226(1.157-1.300) | 1.162(1.095-1.232) |
| **ALD** | 2.082(1.962-2.209) | 1.836(1.729-1.949) | 1.478(1.392-1.57) | 1.579(1.486-1.677) |

^a^Adjusted for age and sex. ^b^Adjusted for age, sex, income, smoking status, regular physical activity, and CCI score. ^c^Adjusted for age, sex, income, smoking status, regular physical activity, type 2 diabetes mellitus, hypertension, dyslipidemia, chronic kidney disease, chronic heart disease, chronic lung disease, and immunocompromised status.

Abbreviations, ALD, alcohol-related liver disease; CCI, Charlson comorbidity index; CI, confidence interval; HR, hazard ratio; IR, incidence rate; MASLD, metabolic dysfunction-associated steatotic liver disease; MetALD, metabolic dysfunction and alcohol-related liver disease; Ref., reference, SLD, steatotic liver disease.
